# Mitochondrial DNA Release in Innate Immune Signaling

**DOI:** 10.1146/annurev-biochem-032620-104401

**Published:** 2023-03-31

**Authors:** Laura E. Newman, Gerald S. Shadel

**Affiliations:** Salk Institute for Biological Studies, La Jolla, California, USA

**Keywords:** disease, DNA sensing, inflammation, mitochondria, pore

## Abstract

According to the endosymbiotic theory, most of the DNA of the original bacterial endosymbiont has been lost or transferred to the nucleus, leaving a much smaller (~16 kb in mammals), circular molecule that is the present-day mitochondrial DNA (mtDNA). The ability of mtDNA to escape mitochondria and integrate into the nuclear genome was discovered in budding yeast, along with genes that regulate this process. Mitochondria have emerged as key regulators of innate immunity, and it is now recognized that mtDNA released into the cytoplasm, outside of the cell, or into circulation activates multiple innate immune signaling pathways. Here, we first review the mechanisms through which mtDNA is released into the cytoplasm, including several inducible mitochondrial pores and defective mitophagy or autophagy. Next, we cover how the different forms of released mtDNA activate specific innate immune nucleic acid sensors and inflammasomes. Finally, we discuss how intracellular and extracellular mtDNA release, including circulating cell-free mtDNA that promotes systemic inflammation, are implicated in human diseases, bacterial and viral infections, senescence and aging.

## THE EVOLUTION OF MITOCHONDRIAL DNA RELEASE

Since its discovery in the early 1960s, studies of mitochondrial DNA (mtDNA) have led to many surprises, ranging from unique modes of gene expression and replication to its role in the tissue-specific pathology of maternally inherited diseases and aging. Here, we review the latest exciting chapter in the mtDNA story: its direct involvement in innate immune signaling and inflammation due to, of all things, escape from its normal habitat in the mitochondrial matrix. At face value, this function of mtDNA is seemingly unlinked to its critical and well-known function in oxidative phosphorylation (OXPHOS) via encoding critical proteins of the mitochondrial electron transport chain and ATP synthase. Interestingly, the systematic study of mtDNA escape began in the early 1990s, when investigations in budding yeast, *Saccharomyces cerevisiae*, by Thorsness and Fox (1, 2) revealed for the first time not only that mtDNA can escape from the mitochondrial matrix to the nucleus, but that nuclear gene mutations can increase the frequency at which this occurs. At the time, these yeast mitochondrial escape (*YME*) genes were interpreted in terms of the known paring down and transfer of the original bacterial DNA to the nucleus during the evolution of the eukaryotic genome. However, they are now appreciated to also be involved in the release of mtDNA from mitochondria for innate immune signaling. For example, the mammalian homolog of yeast Yme1p, YME1L (EC 3.4.24.B18), a mitochondrial inner membrane protease involved in coordinating mitochondrial dynamics with the metabolic state of the cell, is also involved in regulating the balance of nucleotides between the cytoplasm and mitochondria. Loss of this function causes mtDNA release and innate immune signaling (3). Likewise, knock-out of the yeast Yme3p homolog, VPS13C, which forms a lipid transfer channel between the endoplasmic reticulum (ER) and lysosomes, causes mtDNA release downstream of lysosome stress and aberrant innate immune activation (4). Interestingly, mutations in VPS13C (aka PARK23) cause familial, early-onset Parkinson’s disease, adding to the mounting evidence that mtDNA-mediated innate immune activation is involved in this disease (4–7). These examples, and the fact that a variety of gene mutations affect the rate of mtDNA escape in yeast, indicate that multiple processes and pathways can impinge on mtDNA release and that this is cell-type and physiological-context specific. Furthermore, as we discuss in the sections titled Mechanisms of Mitochondrial DNA Release and Mitochondrial DNA Release-Mediated Innate Immune Signaling, mtDNA release and downstream innate immune signaling involve complex dynamics between many organelles in addition to mitochondria (e.g., ER, lysosomes, endosomes) and contribute to inflammatory human disease pathology by multiple mechanisms (8) (Table 1).

## UNIQUE FEATURES OF MAMMALIAN MITOCHONDRIAL DNA AND ITS ORGANIZATION INTO NUCLEOIDS

Mammalian mtDNA is a ~16.5-kb circular molecule that encodes thirteen essential proteins of the mitochondrial OXPHOS system and two rRNAs and 22 tRNAs needed for their translation by a dedicated set of mitochondrial ribosomes (9). In most cells, there are thousands of copies of mtDNA that reside within the matrix of mitochondria and are closely associated with the inner mitochondrial membrane (IMM). Importantly for our discussions, mtDNA is organized into discrete protein–nucleic acid structures called nucleoids that, for all intents and purposes, are the mitochondrial version of chromatin, where replication, repair, transcription, and other steps in mitochondrial gene expression occur and are regulated (10). Nucleoids are also the segregating and inherited units of mtDNA (11). Based on super-resolution microscopy, nucleoids have a diameter of ~100 nm and contain on average ~1.4 molecules of mtDNA (12), which likely equates to a mixture of single, nonreplicating molecules and those at various intermediate stages of replication. The packaging of nucleoids is driven by transcription factor A mitochondrial (TFAM), which binds and bends mtDNA and forms cross-strand dimers that compact the circular molecule. While TFAM is sufficient to drive the full compaction of mtDNA (13, 14), nucleoids contain many other proteins involved in replication, transcription, translation, and other functions (10).

Concerning our coverage of the circumstances that contribute to the release of mtDNA and the state it is in when released, it is important to introduce some of the factors and mechanisms that are needed for the replication and repair of mtDNA. These subjects were the focus of review in this journal by Shadel and Clayton in 1997 (9), making this the ~25th anniversary of that article. Since there are more contemporary reviews on these topics (10, 15), we cover only some relevant highlights here. First, mtDNA replication requires dedicated nucleus-encoded factors, including a minimal replisome consisting of a two-component mitochondrial DNA polymerase γ (EC 2.7.7.7), the DNA helicase TWINKLE (EC 5.6.2.3), and the mitochondrial single-strand DNA-binding protein. Initiation of leading-strand mtDNA replication requires RNA primers that are generated via transcription. Originally, these were reported to emanate from a single so-called light-strand promoter (9, 16), but recently a second light-strand promoter has also been proposed to be a source of replication primers (17). There are at least two topoisomerases required to facilitate transcription and replication of mtDNA (18). For example, one of the final steps of mtDNA replication requires a mitochondrial isoform of topoisomerase 3a (TOP3a) (EC 5.6.2.1) to resolve the two circles that are physically linked as a hemicatenane (19). In terms of DNA repair capacity, mammalian mitochondria have a fairly robust base-excision repair system for oxidative or other non-bulky damage that is initiated by mitochondrial isoforms of several DNA glycosylases, including OGG1 (9, 15). However, they lack many of the other DNA repair pathways available in the nucleus such as nucleotide excision repair and nonhomologous end joining (9, 15). This relative paucity of mtDNA repair pathways, in combination with its high copy number and propensity for damage, led us to propose that mtDNA is a cellular genotoxic stress sentinel that signals, in part, through mtDNA-mediated induction of interferon-stimulated genes (ISGs) that protect the nuclear DNA (20). There are many other factors, including a variety of nucleases, that are involved in mtDNA metabolism (21) that we do not have space to review. However, those relevant to mtDNA release and innate immune sensing are discussed in context.

## MECHANISMS OF MITOCHONDRIAL DNA RELEASE

**Figure 1:**
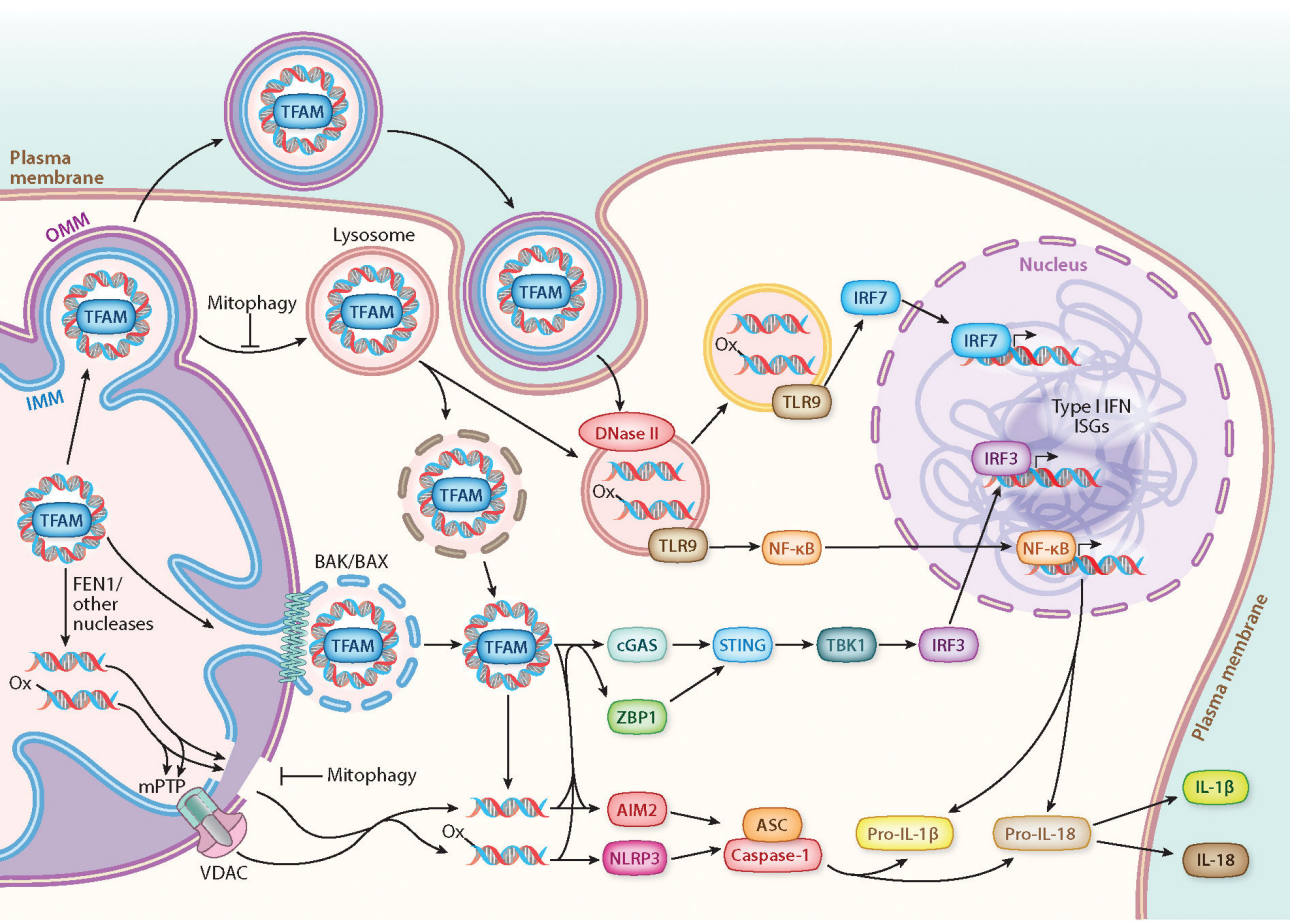
mtDNA-driven innate immune signaling. Release of mtDNA can occur through several different mechanisms, including IMM herniation through BAK/BAX pores in the OMM or escape involving mPTP and VDAC pores. Escape of mtDNA may also occur through leaky mitochondria or lysosomes, arising from defective mitophagy, autophagy, or membrane trafficking. Release of mtDNA into circulation can be achieved through mitochondria-derived extracellular vesicles, in addition to other mechanisms discussed in the section titled Mechanisms of Mitochondrial DNA Release. Released mtDNA has been reported to be whole nucleoids (via BAK/BAX) or fragments (via VDAC), and nonoxidized and oxidized mtDNA (denoted by -ox) signal differently. Cytoplasmic mtDNA activates several innate immune sensors, including (but not limited to) TLR9 (with some specificity for hypomethylated CpG DNA, which includes mtDNA), cGAS, ZBP1, NLRP3, and AIM2 inflammasomes. The activation of these different sensors can trigger a plethora of downstream signaling outcomes, including (but not limited to) ISGs, type I IFN, NF-κB, and the secretion of IL-1β and IL-18. Therefore, there is a wide diversity in mtDNA release mechanisms, species of released mtDNA, and mtDNA-triggered inflammatory pathways that can crosstalk with each other. This complexity likely explains how mtDNA-driven inflammation is tailored for specific cellular stress responses or contributes to tissue-specific pathology. Abbreviations: BAK, Bcl-2 homologous antagonist killer; BAX, Bcl-2-associated X protein; cGAS, cGMP–AMP synthase; IFN, interferon; IL, interleukin; IMM, inner mitochondrial membrane; IRF, interferon response factor; ISG, interferon-stimulated gene; mPTP, mitochondrial permeability transition pore; mtDNA, mitochondrial DNA; NF-κB, nuclear factor κB; NLRP3, NOD-, LRR-, and pyrin domain-containing protein 3; OMM, outer mitochondrial membrane; TBK1, TANK-binding kinase 1; TFAM, transcription factor A mitochondrial; TLR, Toll-like receptor; VDAC, voltage-dependent anion channel; ZBP1, Z-DNA binding protein 1.

**Figure 2:**
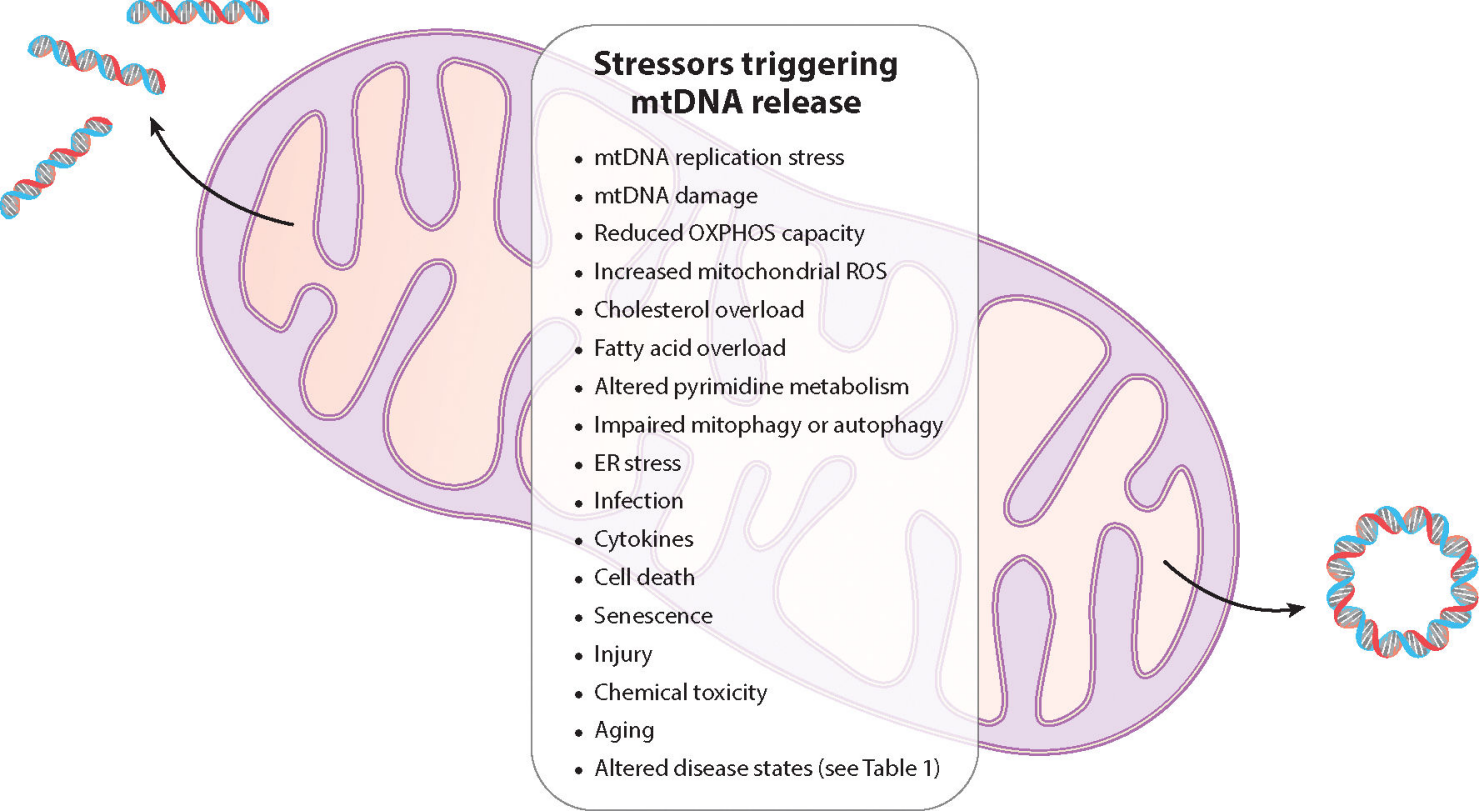
Stressors associated with mtDNA release. Release of mtDNA can be triggered by a variety of stressors. These include problems with mtDNA homeostasis (impaired replication, segregation, or damage), mitochondrial OXPHOS dysfunction (increased ROS production, inhibited electron transport), altered cellular metabolism (cholesterol, fatty acids, or pyrimidines), impaired mitophagy or autophagy, and ER stress. Several stressors that affect the cell and/or organism also cause mtDNA release, and mtDNA-driven inflammation has been linked to several pathological contexts (see Table 1). Abbreviations: ER, endoplasmic reticulum; mtDNA, mitochondrial DNA; OXPHOS, oxidative phosphorylation; ROS, reactive oxygen species.

There are now reports of cytoplasmic and extracellular mtDNA release in many different mammalian cell types and under a variety of physiological and pathological circumstances (Figures 1 and 2) (Table 1). This implies that there are multiple routes of mtDNA escape that operate under context-specific circumstances. Furthermore, it is important to note that released mtRNA has also been reported to activate innate immune RNA sensors (22, 23). Thus, the full range of damage-associated molecular patterns (DAMPs) released from mitochondria and what determines the apparent selectivity of their release remains to be determined. Therefore, we have limited our discussion to the current landscape of mtDNA release mechanisms, while acknowledging that this is a fast-moving area with new mechanistic details steadily coming to light.

### BAK/BAX Pores

Cytoplasmic release of mtDNA via megapores formed by BAK and BAX was originally observed under conditions of caspase-independent apoptosis (24, 25) but has since been observed under multiple other conditions (26–29). Here, the IMM extrudes (or herniates) through the outer mitochondrial membrane (OMM), with the release of entire nucleoids once the pore grows wide enough (Figure 1). The absolute and relative amounts of BAK and BAX determine the rate of growth of the megapore and the kinetics of mtDNA release (30), which is likely a key determinant of the cell-type specificity and sensitivity of the process. How the mtDNA is released into the cytoplasm from this point is not entirely clear. A minority of IMM-derived compartments reportedly have membrane breaks, possibly explaining how mtDNA can escape (24), while others have argued that the IMM becomes more permeable to small ions under caspase-inhibited apoptosis (25). This transition is independent of the well-known mitochondrial permeability transition pore (mPTP), but whether this is sufficient to promote the translocation of mtDNA into the cytoplasm is not known. Whether the whole nucleoid remains intact or is fragmented once in the cytoplasm is also not yet clear.

### VDAC Pores

A second cytoplasmic mtDNA release mechanism involves the coordination of two other mitochondrial pores, voltage-dependent anion channel (VDAC) in the OMM and the mPTP (31) (Figure 1). The precise composition of the mPTP is not known, while the VDAC pore implicated in the mtDNA release process appears to involve pure or mixed oligomers of VDAC1 and VDAC3. The involvement of VDAC2 has not been assessed. During the VDAC-mediated process, mtDNA fragments, not whole nucleoids, are released and this is facilitated under conditions where the mtDNA is oxidatively damaged (31) (Figure 1). This is consistent with early reports that mtDNA fragments of up to 700 bp can be released from isolated mitochondria in an mPTP-dependent manner under conditions of oxidative stress (32), and that oxidized mtDNA (ox-mtDNA) is inflammatory (33). Precisely how the mPTP allows mtDNA release through the IMM remains a mystery, but calcium-dependent and calcium-independent processes have been proposed (31, 34, 35). Once in the intermembrane space, mtDNA binds to the N terminus of VDAC via critical lysine and arginine residues and promotes VDAC oligomerization and mtDNA release (31). However, in macrophages stimulated with inflammasome agonists, mPTP activation and VDAC oligomerization can occur independently of mtDNA binding and reactive oxygen species (ROS)-mediated oxidation (34). In this case, fragments of mtDNA to be released are generated by the FEN1 nuclease (EC 3.1.99.B1) (Figure 1), with another mitochondrial nuclease, MGME1, possibly also involved. This process does not require MRE11, which is needed for mtDNA release in Fanconi anemia cells that are undergoing mtDNA replication fork stress (36), although the mechanism of mtDNA release in that study was not determined and may not be via mPTP and VDAC pores. In mouse lung fibroblasts, release via the mPTP–VDAC mechanism is regulated by binding of vaccinia virus-related kinase 2 to VDAC1 in a kinase-independent manner, which enhances mtDNA binding to VDAC1 (35). This illuminates the interesting possibility that VDAC pore formation for mtDNA release is subject to regulation by other factors that may contribute to the cell specificity of the response.

#### Gasdermin pores.

Gasdermin is an effector molecule of an inflammatory form of cell death called pyroptosis. It is activated by caspase cleavage, producing an N-terminal proteolytic fragment that inserts into the plasma membrane and oligomerizes into large pores that swell and lyse the cell (37). Huang et al. (38) have proposed that an N-terminal fragment generated by caspase-11 (EC 3.4.22.64) cleavage also inserts into mitochondrial membranes to induce mtDNA release in endothelial cells exposed to lipopolysaccharide (LPS). Though intriguing, it remains uncertain whether gasdermin is alone sufficient to allow mtDNA to cross both the IMM and OMM, since it normally forms pores only in the single plasma membrane. Furthermore, gasdermin action at the plasma membrane causes mitochondrial dysfunction that may lead to cytoplasmic mtDNA release by other mechanisms and ultimately extracellular mtDNA release (39).

#### Alterations in autophagy, mitophagy, and lysosomes.

Another emerging, but less well-delineated, mechanism of mtDNA release involves the accumulation of damaged, leaky mitochondria as a consequence of a lack of removal via mitophagy, as well as inefficient autophagy and lysosomes that cannot adequately degrade mtDNA and leak out the excess. For example, Sliter et al. (5) showed that exhaustive exercise in *Parkin* or *Pink1* null mice, or in *Parkin*/Polγ mutator mice, drives inflammation that is presumably due to mtDNA release into the cytoplasm and/or systemically. These data suggest that a lack of PINK1/PARKIN-mediated mitophagy can lead to mtDNA release under extreme mitochondrial stress conditions, a conclusion supported by other studies (40, 41). Although cytoplasmic mtDNA release was not actually demonstrated, and the origin of circulating/systemic mtDNA was not clear, it is logical to postulate that this was due to the accumulation of defective, leaky mitochondria.

In a similar vein to mitophagy, several lines of evidence link defective autophagy and lysosomal function to mtDNA leakage into the cytoplasm (4, 42–44). Impaired autophagy prevents the degradation of mtDNA, enabling its escape from lysosomes (43, 44). Knockout of VPS13C in HeLa cells leads to cytosolic mtDNA release that is associated with defective lysosomes (4). Again, the mechanism of mtDNA release in these studies was not divulged but could be due to the accumulation of leaky mitochondria due to impaired mitophagy or autophagy or to inefficient lysosomes that cannot adequately degrade mtDNA during mitophagy and therefore leak. In addition, nucleoids that do not properly segregate, due to replication stalling or damage, can be extracted from mitochondria and degraded through the endolysosomal system (42, 45). However, mtDNA can escape degradation when this pathway is overwhelmed and get released into the cytoplasm (42).

### Extracellular Mitochondrial DNA Release and Circulating Cell-Free Mitochondrial DNA

In addition to cytoplasmic mtDNA escape, mtDNA can be released from cells passively, due to physical cell damage or injury or during certain immunogenic forms of cell death, or actively, via extracellular vesicles (EVs) or as a component of neutrophil extracellular traps (NETs) (46, 47). Furthermore, mtDNA can gain access to the circulatory system and be detected as so-called circulating cell-free mtDNA (CCF-mtDNA) (48–52). EVs containing mtDNA may arise from mitochondria-derived vesicles (MDVs), given that they frequently contain other immunogenic molecules (53). However, a recent study of MDVs did not identify mtDNA as cargo (54), suggesting that other mechanisms mediate the transfer of mtDNA to EVs or that there may be uncharacterized species of MDVs or other mitochondrial-membrane-derived compartments that carry mtDNA to EVs. PINK1, which regulates mitophagy, also promotes the packaging of mtDNA into EVs, thereby facilitating cellular mtDNA release (55, 56). This function of PINK1 appears separate from its role in mitophagy, as a kinase-dead PINK1 mutant, which cannot activate PARKIN, facilitates mtDNA packaging into EVs, and PARKIN expression prevents packaging of other immunogenic molecules into EVs (53, 56). There remains much to be learned about how EVs interface with mitophagy and membrane trafficking of mitochondrial components, including mtDNA.

## MITOCHONDRIAL DNA RELEASE-MEDIATED INNATE IMMUNE SIGNALING

Cells have evolved intricate systems to detect and defend against invading pathogens. Key to the initial cellular response is a diverse repertoire of pattern-recognition receptors (PRRs) that bind with specificity to various pathogen-associated molecular patterns (PAMPs), which are molecules from bacteria, viruses, and other pathogens that cells recognize as foreign. PRRs include a variety of PAMP-sensing proteins, including Toll-like receptors (TLRs), inflammasomes, RNA sensors of the RIG-I-like receptor (RLR) family, and DNA sensors like cGMP–AMP synthase (cGAS) (EC 2.7.7.86). RLRs, cGAS, and TLRs can activate type 1 interferon (IFN) responses and downstream ISGs via activation of IFNα and IFNβ and/or NF-κB and other signaling pathways to induce proinflammatory cytokines (Figure 1). Alternatively, inflammasomes activate different proinflammatory cytokines [i.e., interleukin (IL)-1β and IL-18] and also promote pyroptosis (57) (Figure 1). Consistent with their bacterial origin, several mitochondrial molecules (including mtDNA, mtRNA, N-formyl peptides, cardiolipin, and TFAM) can bind to PRRs and act as DAMPs, which are endogenous molecules liberated under various stress conditions that activate the innate immune system (8, 58). As we discuss in the sections titled cGAS-STING Signaling, Inflammasome Activation, and TLR9 Signaling, depending on the precise context and cell type, mtDNA release can activate any of these three classes of PRRs (Figure 1).

### cGAS–STING Signaling

A major cellular DNA sensor is cGAS, a member of the nucleotidyltransferase family of enzymes that generates 2′3′ cyclic GAMP (cGAMP) from ATP and GTP (59). STING is an ER membrane-resident protein that binds cGAMP, after which it translocates to the Golgi, where it activates TANK-binding kinase 1 (TBK1) (EC 2.7.11.10). TBK1, in turn, phosphorylates the transcription factor interferon response factor 3 (IRF3) allowing it to translocate into the nucleus and activate the IFNβ gene to kick off a full type 1 IFN and ISG response (60, 61). This entire cascade of events is initiated by the ability of cGAS to bind double-stranded DNA (dsDNA) and undergo higher-order conformational and state changes that activate its enzymatic activity, which is reviewed elsewhere (62). Importantly for our discussions, cGAS is not only activated by exposure to viral DNA but also mis-localized cellular DNA, including both cytoplasmic chromatin fragments and mtDNA (63–67).

#### TFAM depletion and its direct role in cGAS recognition.

The first report of mtDNA release caused by the deficiency of a mitochondrial protein involved directly in mtDNA metabolism was in cells depleted of TFAM (65). Because TFAM is needed for both transcription and replication of mtDNA, complete knockout of TFAM in mice causes embryonic lethality due to loss of mtDNA and OXPHOS (68). Interestingly, heterozygous TFAM knockout (*Tfam*^+/−^) mice are viable despite having 50–70% depletion of mtDNA in most tissues (68). While probing the potential consequences of partial mtDNA depletion in *Tfam*^+/−^ mouse embryonic fibroblasts (MEFs), West et al. (65) found ISGs are the top upregulated gene set, indicating upregulation of innate immune signaling. They went on to show in these cells and in TFAM-depleted bone marrow–derived macrophages that mtDNA is released into the cytoplasm, where it activated the cGAS–STING–TBK1 pathway. Furthermore, *Tfam*^+/−^ MEFs and mice are resistant to viral infection, demonstrating that mtDNA release can prime an antiviral innate immune response. The cells have elongated mitochondria and enlarged nucleoids, suggesting that inhibited mitochondrial fission downstream of an mtDNA replication defect is driving the release of mtDNA (42). Interestingly, bent, TFAM-bound DNA is a preferred substrate of cGAS (69), which indicates that cGAS activation by mtDNA is facilitated by the concomitant release of bound TFAM (Figure 1). This is also consistent with TFAM-bound nucleoids being released in *Tfam*^+/−^ cells or through BAK/BAX pores during apoptotic signaling (24, 25, 42). Recently, a physiologically relevant context in which TFAM depletion drives mtDNA-release-mediated cGAS–STING signaling in humans was elucidated (70). In macrophages with reduced expression of TET2 (EC 1.14.11.n2) or DNMT3A (EC 2.1.1.37), which causes clonal hematopoiesis and increased risk for atherosclerosis, TFAM expression is reduced, driving mtDNA release. Interestingly, this is due to loss of direct binding of TET2 and DNMT3A to the TFAM promoter and recruitment of activating transcription factors, a function independent of their enzymatic activities. Reduced TFAM expression may also play a direct role in mtDNA-release-mediated cGAS–STING activation that contributes to kidney inflammation and fibrotic disease (71) and is observed in cancer cells with decreased ATM expression or activity (72).

#### Other forms of mitochondrial DNA replication stress.

The TFAM paradigm suggested that other forms of mtDNA stress might also lead to mtDNA-release-mediated cGAS–STING signaling, which has turned out to be the case. Cells either lacking TOP1MT (EC 5.6.2.1) or expressing a pathogenic variant associated with lupus exhibit mtDNA release and activation of the cGAS–STING pathway (73). TOP1MT knockout cells have reduced mtDNA copy number and mitochondrial transcripts, defects in nucleoid organization, elongated mitochondria, and lower OXPHOS. While mtDNA depletion is rescued by expression of the pathogenic variant, the OXPHOS defect is not, and the remaining phenotypes, including mtDNA release, are only partially rescued. Thus, this form of mtDNA and cGAS–STING activation may be involved in lupus pathology. In certain Friedreich’s ataxia patient cells, there is a defect in mtDNA fork protection during replication that leads to mtDNA-release-mediated cGAS–STING activation in a manner that requires MRE11 (36). Loss of the mitochondrial CLPP protease (EC 3.4.21.92) causes mtDNA instability and nucleoid alterations that promote release of mtDNA by a VDAC-dependent mechanism resulting in cGAS–STING activation (74). While the precise role of CLPP in mtDNA maintenance is not known, mtDNA-release-mediated cGAS–STING signaling may be involved in Perrault syndrome caused by CLPP mutations. As mentioned in the section titled The Evolution of Mitochondrial DNA Release, knockout of YME1L results in mtDNA release in a VDAC-dependent manner and cGAS–STING activation (3). Here, the effect is mediated by stabilization of the mitochondrial pyrimidine carrier SLC25A33. Increased SLC25A33 activity depletes cytoplasmic pyrimidine pools by increasing their transport into mitochondria. This influx of nucleotides increases de novo synthesis of mtDNA and/or creates some type of replication stress that leads to mtDNA release. This situation is reminiscent of another involving cytidine/uridine monophosphate kinase 2 (CMPK2) (EC 2.7.4.14), a mitochondrial pyrimidine kinase that is increased in macrophages stimulated with LPS, leading to an increase in de novo mtDNA synthesis and mtDNA release via a VDAC-dependent mechanism (75). Therefore, an interesting connection between mitochondrial and cytoplasmic pyrimidine metabolism and mtDNA-mediated innate immune signaling is clearly emerging. Deletion of the mitochondrial nuclease ENDOG (EC:3.1.30) causes ROS-dependent release of small mtDNA fragments (100–200 bp) via VDAC pores and cGAS–STING activation (31). This was the first report of VDAC pore–mediated mtDNA release and mtDNA–cGAS–STING innate immune signaling in lupus (31). However, the nature of the mtDNA defect caused by the loss of ENDOG, the precise role of ROS, and the origin of the released mtDNA fragments (e.g., which nucleases or processes generate them) remain to be clarified.

#### Altered nucleoid–membrane interactions and endoplasmic reticulum interactions.

Nucleoid clustering and disorganization are frequently documented vis-à-vis mtDNA release, highlighting a role for the IMM in restraining innate immune signaling via proper nucleoid maintenance. As already mentioned, mtDNA interacts with the IMM via the MICOS/MIB complexes and cholesterol. Deletion of SAMM50, a component of the MIB complex, causes nucleoid clustering and release of whole nucleoids in a BAK/BAX-dependent fashion (26). BAK/BAX pore formation and mtDNA release were dependent upon the synthesis of cardiolipin, a mitochondrial lipid that is important for cristae curvature, as well as the transport of cardiolipin to the OMM (26). Notably, the MICOS complex binds cardiolipin, and depletion of the core MICOS subunit MIC60 also caused nucleoid clustering (26). Therefore, SAMM50 acts through MICOS to prevent cardiolipin externalization and mtDNA escape, thereby highlighting the importance of IMM integrity to mtDNA disorganization and mtDNA release (26). Loss of ATAD3A also causes mtDNA release through VDAC pores (76). ATAD3A regulates IMM morphology, presumably by maintaining domains of cholesterol at IMM/OMM boundary sites. ATAD3A deletion causes IMM disorganization (77) and prevents membrane association of the replicating nucleoid (78), again pointing to a role for IMM morphology in preventing mtDNA disorganization and release. ATAD3 was also reported to interact with the D-loop region of mtDNA (79), suggesting this interaction may also contribute to improper nucleoid localization and mtDNA release. Consistent with this line of evidence, ATAD3 coordinates with SAMM50 and TWINKLE to extract damaged mtDNA from mitochondria for elimination by lysosomes (45). Lastly, since mtDNA replication occurs at IMM/OMM boundary sites within close vicinity of mitochondria–ER contact sites, the ER is also linked to mtDNA release. Mitochondria–ER contacts license mtDNA replication as well as segregation, and wholesale perturbation of ER morphology causes enlarged nucleoids, consistent with a role for the ER in nucleoid organization (80). ER stress induces mitochondrial dysfunction and stimulates mtDNA release, leading to the activation of cGAS as well as the NLRP3 inflammasome (81, 82).

#### Apoptosis and related signaling events.

It is well established that mitochondria are critical for cell death signaling, including regulating cell-intrinsic apoptosis via the release of various proapoptotic factors (e.g., cytochrome c) after mitochondrial outer membrane permeabilization (MOMP). However, two seminal studies demonstrated that mtDNA is also released during apoptosis and activates cGAS–STING signaling (63, 64). Unveiling this pathway required activating apoptosis under conditions of caspase inhibition, suggesting that one function of caspase activation is to keep apoptosis immunologically silent. Apoptosis and mtDNA release under these conditions requires BAK and BAX activation (63, 64), and it was subsequently shown for the first time that mtDNA escapes though BAK/BAX megapores (24, 25). More recently, the phenomenon of minority-MOMP has been described through which sublethal stress can cause the permeabilization of only a few mitochondria without inducing apoptosis, which could be the more physiological mechanism through which mtDNA is released via BAK/BAX pores for innate immune signaling (47, 83).

#### Altered lipid metabolism.

Mitochondria play an important role in lipid synthesis, which occurs at mitochondria–ER contact sites and the IMM. While still a developing area, many interesting new connections between lipid metabolism and mtDNA-release-mediated cGAS–STING signaling have been reported. As already discussed, cholesterol domains are important for IMM structure, as well as mtDNA replication, segregation, and nucleoid organization. Disruptions in cholesterol homeostasis cause nucleoid disorganization (84), and mitochondrial cholesterol overload triggers mitochondrial dysfunction and mtDNA release (85). Cholesterol metabolism is also linked to cGAS–STING and IFN signaling. Induction of type I IFN signaling decreases cholesterol synthesis, and increasing cholesterol via synthesis or supplementation dampens IFN signaling and antiviral resistance (86, 87). In addition, repressing cholesterol synthesis heightens type I IFN signaling by increasing the activity of the cGAS–STING pathway (86). Several players within the cholesterol synthesis pathway also directly regulate STING activity, including its trafficking, ability to activate TBK1–IRF3, and degradation via lysosomes (88, 89). Fatty acid metabolism is also emerging as a key regulator of mtDNA-release-mediated activation of cGAS–STING signaling. Loss of FABPB5, a chaperone important for fatty acid uptake, inhibits fatty acid elongation and desaturation and lowers cardiolipin levels in regulatory T cells (Tregs) (90). This causes mitochondrial dysfunction and mtDNA-release-mediated cGAS–STING activation, leading to increased Treg suppressive capacity (90). Others have reported that palmitic acid overload and lipotoxicity induce mtDNA release and cGAS–STING signaling (91–94), and mtDNA–STING driven inflammation is implicated in the pathology of several high-fat diet (HFD) mouse disease models (Table 1) (92, 93, 95, 96). Thus, the intersection of lipid metabolism, mtDNA release, and cGAS–STING signaling will be an exciting area going forward.

#### Other stimuli.

While the number of reports of conditions under which mtDNA is released is at this point too vast to summarize in this article due to space limitations, a sampling is provided here, and general categories are listed in Figure 2. This includes exposure of cells to several chemicals and drugs, including eribulin mesylate (anticancer microtubule destabilizer) (97), rocaglamide (anticancer translation and autophagy inhibitor) (98), chitosan (vaccine adjuvant) (99), and 2-chloroethyl sulfide (toxin related to mustard gas) (100). Exposure to the cytokines IL-6, IL-1β, and TNF also leads to mtDNA-release-mediated cGAS signaling in some cell types and contexts (101–103). CD47-blockade-mediated mtDNA–cGAS–STING activation in dendritic cells might be able to be leveraged for immunotherapy approaches (104). LPS treatment of Kupffer cells induces mtDNA release and STING signaling in a DRP1-dependent manner, leading to cell death and liver injury (105). Additionally, reduced dicer expression or Alu–RNA accumulation leads to mtDNA–cGAS–STING signaling in retinal pigment epithelial cells that is part of a noncanonical form of inflammasome activation (106).

### Inflammasome Activation

**Figure 3:**
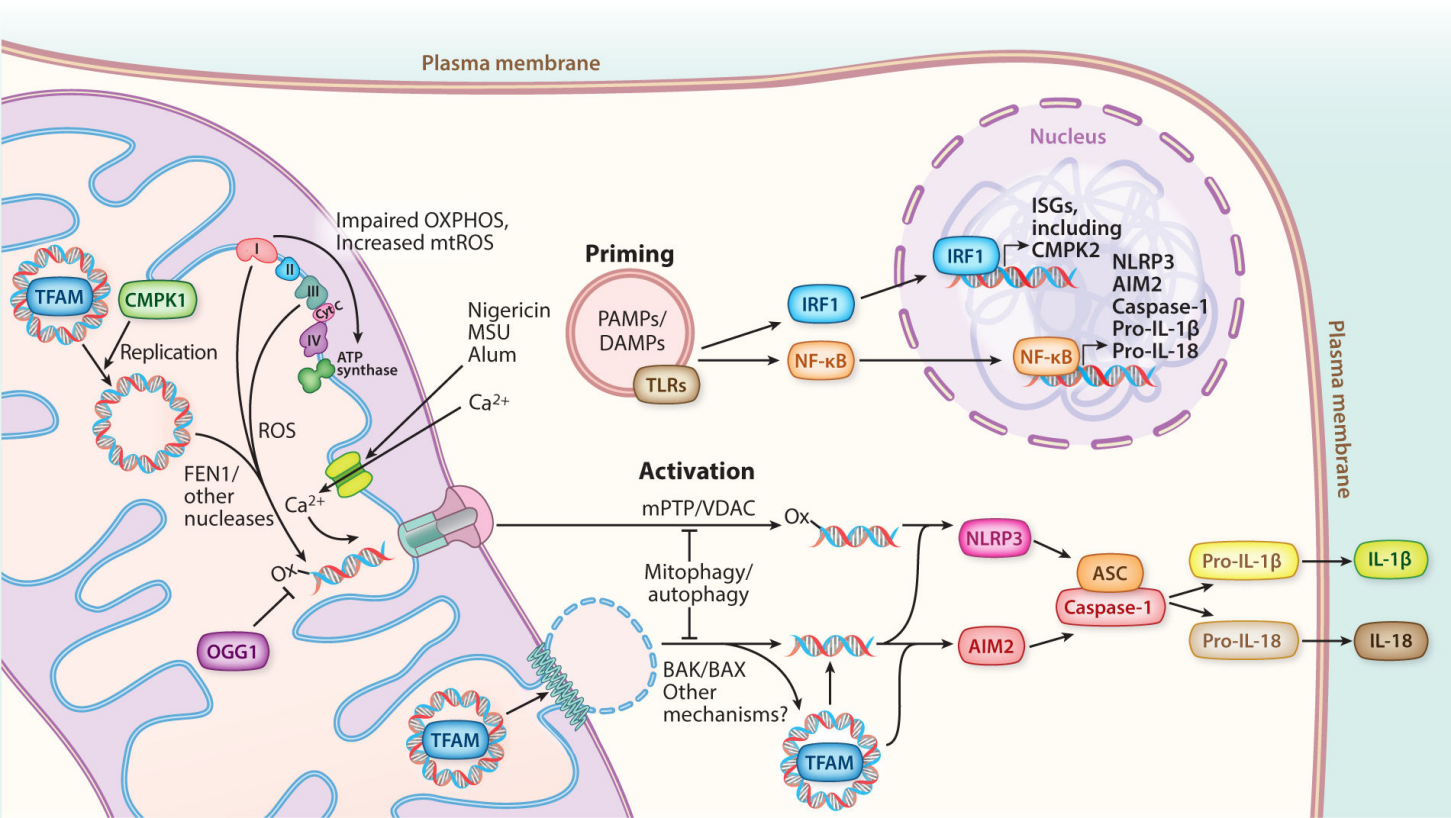
mtDNA-driven inflammasome signaling. Inflammasome signaling requires an initial priming step. PAMPs and DAMPs activate TLRs within the endosomal compartment, initiating signaling cascades that culminate in the expression of genes needed for inflammasome assembly, mediated by NF-κB in the nucleus. In parallel, ISGs are induced, including CMPK2, a mitochondrial nucleotide kinase that increases the rate of new mtDNA synthesis. Increased ROS associated with dysfunctional OXPHOS causes the accumulation of oxidized mtDNA (denoted by -ox), which outpaces the ability of OGG1 to excise 8-oxoguanine. FEN1 and likely other mitochondrial nucleases then process mtDNA into fragments. Conditions that also trigger mitochondrial calcium uptake via the mitochondrial calcium uniporter promote mPTP opening. Activation of inflammasomes is enabled by the released cytoplasmic mtDNA. Oxidized mtDNA fragments are released through mPTP/VDAC pores and activate the NLRP3 inflammasome. BAK/BAX pores, and possibly other mechanisms, enable release of mtDNA (possibly as either fragments or whole nucleoids), which can then bind and activate AIM2. Fully assembled inflammasomes enable caspase-1 to cleave pro-IL-1β and/or pro-IL-18, enabling secretion of the mature cytokines and downstream signaling. Abbreviations: BAK, Bcl-2 homologous antagonist killer; BAX, Bcl-2-associated X protein; CMPK, cytidine/uridine monophosphate kinase; cyt C, cytochrome c; DAMP, damage-associated molecular pattern; IL, interleukin; IRF, interferon response factor; ISG, interferon-stimulated gene; mPTP, mitochondrial permeability transition pore; mtDNA, mitochondrial DNA; NF-κB, nuclear factor κB; NLRP3, NOD-, LRR-, and pyrin domain-containing protein 3; OXPHOS, oxidative phosphorylation; PAMP, pathogen-associated molecular pattern; ROS, reactive oxygen species; TFAM, transcription factor A mitochondrial; TLR, Toll-like receptor; VDAC, voltage-dependent anion channels.

In addition to DNA sensors like cGAS–STING and Z-DNA binding protein 1 (ZBP1), which largely control transcriptional inflammatory responses, there are inflammasome-associated DNA sensors that primarily drive posttranscriptional inflammatory responses (57). At this point, there are many reports of mtDNA release mediating inflammasome activation. Due to space limitations, we have focused on the literature describing mtDNA-release-mediated NLRP3 and AIM2 inflammasome activation (Figure 3).

#### NLRP3.

NLRP3 (NOD-, LRR-, and pyrin domain-containing protein 3) is a cytosolic innate immune sensor that, in response to multiple PAMPs, DAMPs, and environmental irritants, forms inflammasomes that activate caspase-1 (EC 3.4.22.36) for cleavage of pro-IL-1β and pro-IL-18 into the mature proinflammatory cytokines and to process gasdermin to initiate pyroptosis (107). Like most inflammasomes, NLRP3 activation requires an initial TLR-dependent and NF-κB-dependent priming step, to transcriptionally induce the genes encoding NLRP3, caspase-1, and pro-IL-1β, and initiate other posttranscriptional events (Figure 3). However, unlike most inflammasomes, NLRP3 is activated by many different types of seemingly unrelated stimuli. We (8, 58) and others (108) have reviewed previously the early work on how mitochondrial stress is an important, and perhaps the common, feature of NLRP3 inflammasome activation. This includes the seminal work showing that lack of mitophagy causes ROS and mtDNA release and that these conspire to promote this pathway (44, 109). Therefore, here, we summarize some of the more recent developments on the role of mtDNA, and in particular ox-mtDNA, in NLRP3 inflammasome activation.

There is now substantial evidence that mtDNA, and in particular ox-mtDNA, is a bona fide activator of the NLRP3 inflammasome under many different conditions. Consistent with the original proposal of Shimada et al. (109) that ox-mtDNA is the culprit in NLRP3 inflammasome activation, macrophages lacking the base-excision repair enzyme OGG1, which cannot repair oxidative purine damage (e.g., 8-oxoguanine) in mtDNA and nuclear DNA, show increased release of ox-mtDNA and NLRP3 inflammasome activation that is partially rescued by expressing OGG1 purposefully targeted to mitochondria (110). These authors also provide evidence consistent with ox-mtDNA driving NLRP3-dependent inflammation in mouse models of atherosclerosis. In atherosclerosis, there may also be a feedforward loop whereby mtDNA-driven NLRP3 activation of caspase-1 inhibits PARKIN-meditated mitophagy, which leads to the accumulation of additional mitochondrial damage and ox-mtDNA release (111). However, under non-atherogenic circumstances, NF-κB activates PARKIN/p62-mediated mitophagy to restrain excessive NLRP3 hyperactivation (112), and there is also communication between the endosomal pathway and mitophagy via the adaptor APPL1 that attenuates mtDNA release and NLRP3 activation (113). NLRP3 activation also causes localization of the SHP2 phosphatase (EC 3.1.3.48) to mitochondria, where it dephosphorylates ANT1 to prevent additional mPTP-mediated mtDNA release and NLRP3 hyperactivation (114). Additional recent evidence is consistent with various stressors leading to an accumulation of ox-mtDNA that activates NLRP3: Knockout of TRX2 (thioredoxin 2) debilitates a major mitochondrial antioxidant pathway in brown adipose tissue (115); diabetic murine and palmitate-conditioned murine peritoneal macrophages have defects in mitophagy due to reduced FOXO3a (EC 2.7.11.1)-driven PINK1 transcription (116); oxygen/glucose deprivation and reperfusion of BV2 increases mitochondrial ROS in microglial and PC12 neuronal cells (117); Kupffer cells experience oxidative stress during in vivo and in vitro ischemia/reperfusion injury (118); influenza infection of macrophages (119) and severe fever with thrombocytopenia syndrome virus (SFTSV) infection of THP-1 cells trigger release (120); induction of cyclooxygenase-2 (121) or nickel toxicity (122) in macrophages exacerbates mitochondrial damage and ROS; and depletion of MRE11a in T cells causes mtDNA release associated with impaired mitochondrial function (123). There are also several conditions reported to inhibit mtDNA-release-mediated NLRP3 activation that all point to reducing mitochondrial ROS directly or via inhibiting OXPHOS as the cause of inhibition, consistent with ox-mtDNA as the activating signal. This includes exposure of macrophages to molecular hydrogen (124), carbon monoxide (125), ethyl pyruvate (126), dimethyl fumarate (127), or metformin (128). Interestingly, acetylcholine also inhibits mtDNA release and NLRP3 activation in macrophages, suggesting a novel neurotransmitter–immune cell anti-inflammatory signaling pathway (129). In addition, the tumor suppressor PML promotes mitochondrial ROS production, mtDNA release, and NLRP3 activation (130). In summary, the complicated mix of factors that control mtROS production and accumulation and ox-mtDNA release may begin to explain how NLRP3 activation is responsive to so many different stimuli and operates under many physiological and pathological stress conditions.

Detailed mechanistic studies in macrophages have illuminated a novel connection between increased mtDNA replication and release of ox-mtDNA that activates NLRP3. Karin and colleagues (75) found that TLR signaling in macrophages induces IRF1-mediated activation of the mitochondrial nucleotide salvage enzyme, CMPK2, which promotes an increase in mtDNA synthesis (Figure 3). They proposed that the newly synthesized (and hence unpackaged and unprotected) mtDNA is more prone to oxidative damage, providing more ox-mtDNA for release and NLRP3 activation. They went on to show that downstream of several NLRP3 activators that promoted mitochondrial calcium uptake, the endonuclease FEN1 is required to generate fragments of ox-mtDNA that are released from mitochondria in an mPTP- and VDAC-pore dependent manner (34). Interestingly, this also led to cGAS–STING activation and extracellular mtDNA release. Consistent with this model, myeloid-specific deletion of CMPK2 reduced pathology in a mouse model of acute respiratory distress syndrome, suggesting a role for ox-mtDNA-mediated NLRP3 activation in this disease (34). Although it is now clear that ox-mtDNA is a major activating signal for the NLRP3 inflammasome, NLRP3 itself lacks any predicted or empirically defined DNA-binding domain. Thus, exactly how ox-mtDNA engages NLRP3 and whether other DNA-binding components of the system exist is an open question.

#### AIM2.

AIM2 is a key sensor of cytosolic DNA that leads to inflammasome activation. It has a C-terminal HIN (hematopoietic interferon-inducible nuclear protein) domain that binds to dsDNA but is held in an autoinhibited state through intermolecular interactions between its HIN and pyrin domains (131, 132). DNA binding liberates the AIM2 pyrin domain, allowing it to interact with ASC and promoting the subsequent CARD–CARD interactions with procaspase-1 that assemble an active inflammasome complex. In some contexts, AIM2 activation also requires a type-1 IFN response mediated by other DNA sensors (57). As predicted from its ability to bind dsDNA from bacteria and other pathogens, AIM2 can also detect cytoplasmic host DNA, including mtDNA. Interestingly, one of the first indications that mtDNA can activate the AIM2 inflammasome was a study showing bee venom can lead to cytoplasmic mtDNA release in keratinocytes due to one of its components, melittin, which damages mitochondrial membranes (133). Subsequently, in macrophages with altered cholesterol metabolism due to knockout of the gene encoding cholesterol-25-hydroxylase, an anti-inflammatory ISG, there is mitochondrial OXPHOS dysfunction and release of mtDNA into the cytoplasm that activates the AIM2 inflammasome (85). Impaired mitophagy in the hearts of a type II diabetes mouse model is associated with mtDNA-release-mediated AIM2 activation in cardiomyocytes and macrophages after myocardial infarction (134). Similarly, HFD-induced liver AIM2 activation, due to free-fatty-acid overload, also has been reported to be driven, in part, by mitochondrial damage-mediated mtDNA release, which can be suppressed by overexpression of the TGFβ-family cytokine, GDF15 (135). Also in a potentially related process in liver, even though AIM2 was not implicated per se, the mitophagy receptor FUNCDC1 prevents mtDNA-release-mediated inflammasome activation, inflammation, and hepatocellular carcinoma in mice (136). The proteasome inhibitor, epoxomicin, causes mtDNA release in retinal pigment endothelial cells that may drive the AIM2 inflammasome activation observed under these conditions; however, the strict requirement for mtDNA was not tested (137). Perfluorooctane sulfonate, a representative of a class of widely used industrial chemicals, causes mtDNA-release-mediated AIM2 activation, which may underlie its proinflammatory toxicity (29). In that study, mtDNA release occurred via BAK/BAX pores, and AIM2 activation required non-oxidized mtDNA, as opposed to the oxidized mtDNA that activated NLRP3 (29). This likely explains why AIM2 does not interact with endogenous mtDNA under NLRP3-activating conditions (109) but does bind to and is activated by endogenously added mtDNA (44, 109), but of course other factors (e.g., how the DNA is packaged or localized during and after release) could also be important. At this point, most of the examples of mtDNA-mediated AIM2 activation involve harsh stress conditions in which mitochondria are damaged and AIM2 is inappropriately activated. Whether AIM2 also senses mtDNA under less stressful physiological conditions remains to be determined. In this regard, an inflammasome-independent role for AIM2 in regulating mitochondrial fusion in cancer cells has been described (138), which is interesting given the known connections between mitochondrial dynamics and mtDNA replication and maintenance (65, 80).

### TLR9 Signaling

TLRs are a family of ten innate immune sensors that are primarily expressed within innate immune cells and recognize a wide variety of PAMPs and DAMPs (139). TLRs are trafficked to endosomes and the plasma membrane via the ER and can recognize PAMPs or DAMPs from either the extracellular environment or within endosomes during degradation. TLR9 recognizes DNA within the endosome that is enriched for hypomethylated CpG motifs, which includes mtDNA (46, 47). DNases generate degradation products that are ligands for TLR9, with DNase I (EC 3.1.21.1) and DNase 1L3 digesting DNA before internalization and DNase II (EC 3.1.22.1) acting from within acidified compartments. In contrast to cGAS, which is activated by longer, looping fragments of DNA, TLR9 recognizes DNA degradation products. This difference is highlighted by the fact that *Dnase2a*^−/−^ mice display increased inflammation that is driven by STING and not TLR9, suggesting that DNA that escapes endosomal degradation can leak into the cytosol (140, 141). Upon binding to a ligand, TLR9 dimerizes and activates MYD88, leading to NF-κB signaling. Further trafficking of TLR9 to a lysosome-related organelle allows for activation of IRF7 and type-I IFN signaling (142).

We previously reviewed the early key studies that showed that mtDNA can activate TLR9 signaling and inflammation (8), so we do not reproduce that here due to space limitations. Suffice it to say that there is now a vast literature demonstrating that mtDNA can activate TLR9 when it is released from cells and/or into circulation via cell injury, NETs, or EVs, thereby driving local and systemic inflammatory responses (46, 47). Dysfunctional mitophagy and autophagy can also cause TLR9 activation by shuttling mtDNA into lysosomal compartments, which are capable of activating TLR9 cells autonomously (40, 41, 43). Beyond membrane trafficking, other factors can facilitate mtDNA–TLR9 signaling by binding to mtDNA, including TFAM, which is also a DAMP (143–145). An interesting future direction is to determine whether other mitochondrial DAMPs besides TFAM that are present in EVs (53) can also modulate TLR9 or other PRRs. Mounting evidence also indicates that budding of vesicles from mitochondria enables the escape of mtDNA via EVs (53, 146), which then trigger TLR9 signaling when internalized by the endocytic pathway of recipient cells. In addition, mtDNA-TLR9 signaling has been implicated in many inflammatory pathological states (Table 1).

### ZBP1 and Other DNA Sensors

ZBP1 (also known as DAI and DLM-1) was one of the first cellular nucleic-acid sensors discovered (147). ZBP1 is a member of a family of related proteins that share one or two copies of the Z-DNA-binding domain, which includes the RNA-editing enzyme ADAR (EC 3.5.4.37) and the protein kinase PKZ, all of which are involved in pathogen defenses (148). While originally described as a DNA sensor, ZBP1 is now appreciated to also bind and sense viral and endogenous double-stranded and Z-RNAs, including within the nucleus. ZBP1 also binds large helical RNP structures generated during influenza virus infection. ZBP1 contains two RHIM domains that are important for cell death pathways involving RIP kinases 1 and 3 (e.g., necroptosis) and inflammatory responses, including activation of NF-κB. There are several reports of released mtDNA binding ZBP1. In response to hydrogen peroxide produced by the addition of glucose oxidase to cultured lung epithelial cells, ox-mtDNA is released and binds ZBP1 (149), which subsequently activates TBK1–IRF3 signaling. This also results in the extracellular release of mtDNA fragments via exosomes that have paracrine inflammatory effects on naïve epithelial cells in culture. Similar results were reported in retinal pigment epithelial cells (150). In mouse breast cancer cells, glucose deprivation leads to mtDNA release, binding to ZBP1, and RIP1K-independent necroptosis (151). HT29 cells and MEFs undergoing necroptosis release mtDNA in a p53 upregulated modulator of apoptosis (PUMA)-specific manner, which activates both ZBP1 and STING (152). In addition, other innate immune sensors have been implicated in mtDNA sensing that cannot be covered in detail due to space limitations. For example, deletion of IRGM1 in mice results in defective mitophagy with activation of cGAS–STING in fibroblasts but activation of TLR7 in macrophages (153). Interestingly, TLR7 was shown previously to enhance mtDNA release in neutrophils in the context of lupus (154). Likewise, the DNA sensor IFI16 detects released mtDNA in cellular models of Parkinson’s disease (PD) and induces a mixed inflammatory response (6). These examples emphasize that we currently do not know the full cast of innate immune sensors that can respond to released mtDNA under various circumstances, an area that clearly deserves more attention.

## MITOCHONDRIAL DNA RELEASE IN INFECTION, AGING, AND DISEASE

### Release During Viral Infection

Release of mtDNA and activation of antiviral responses occurs as part of the warfare between viruses and cells. Activation of cGAS–STING signaling by released mtDNA provides broad viral resistance (63, 65). Thus, cytosolic mtDNA release can prime antiviral innate immune signaling. The herpes simplex DNA viruses HSV-1 and HSV-2 encode a nuclease, UL12.5, that localizes to the mitochondrial matrix upon infection and depletes mtDNA (155, 156), indicating that depletion of mtDNA may benefit viral replication. However, infection by HSV-1 or expression of UL12.5 causes mtDNA nucleoid clustering and mitochondrial elongation transiently before the complete degradation of mtDNA. In other words, the same mtDNA stress and release pathways active in response to TFAM depletion are also activated during HSV-1 infection (42, 65). Infection by UL12-deficient HSV-1 strains results in reduced cGAS–STING signaling and infection compared to wild-type HSV-1, strongly suggesting that cytosolic mtDNA release activates cGAS–STING signaling during HSV-1 infection and provides antiviral resistance in vivo (65). We have since found that HSV-1 UL12.5 promotes endosomal extraction of enlarged nucleoids, further phenocopying TFAM deficiency and strongly implicating this mtDNA release mechanism during HSV-1 infection (42). Why HSVs encode an apparently antiviral protein that causes mtDNA release is not clear, but other viruses have now been shown to also cause mtDNA release and cGAS activation (Table 1). For example, this has been documented with dengue (DENV), porcine reproductive and respiratory syndrome virus (PRRSV), influenza A virus (IAV), and encephalomyocarditis virus (ECMV) infection, with enlarged nucleoids observed in the latter two cases (157–159). Infection of macrophages with IAV triggers the release of ox-mtDNA and activation of NLRP3 and AIM2 inflammasomes (119). In dendritic cells, the release of ox-mtDNA during DENV infection leads to TLR9 activation, spurred by ROS, mPTP opening, NLRP3 inflammasome activation, and reduction of mtDNA bound to TFAM (159). In cultured cells, mtDNA-driven activation of cGAS restricts DENV replication, and the DENV protein NS2B promotes the degradation of cGAS to facilitate its replication (158). Similarly, PRRSV infection triggers mtDNA–cGAS signaling, which restricts viral replication (160). Infection by Zika virus (ZIKV), measles virus (MeV), or severe acute respiratory syndrome coronavirus 2 (SARS-CoV-2) also causes mtDNA release and cGAS–STING signaling (161–163). SARS-CoV-2 infection also causes mtDNA release and cGAS–STING signaling in endothelial cells (163), as well as TLR9 activation (164). While it is appreciated that mtDNA-driven antiviral signaling can restrict viral replication, overactivation of mtDNA-associated inflammatory responses can also drive pathological inflammation associated with viral infection (120, 163, 164), supported by the observation that circulating mtDNA is associated with severe COVID-19 (165).

An interesting observation from the above studies is the large number of RNA viruses (IAV, DENV, PRRSV, ZIKV, ECMV, MeV, SARS-CoV-2) that stimulate mtDNA release and cGAS–STING signaling (Table 1). Therefore, mtDNA appears to have antiviral signaling properties against RNA viruses in addition to DNA viruses. Though cGAS senses cytosolic DNA, activation of the cGAS–STING pathway restricts the replication of RNA viruses in addition to DNA viruses (160–162). Both DENV and ZIKV promote the degradation of cGAS, apparently to limit antiviral responses initiated by mtDNA (158, 161). How RNA viral infection stimulates mtDNA release is still unclear; however, it is worth noting that MAVS, the adaptor required for dsRNA-driven antiviral responses, localizes to the outer mitochondrial membrane (166). Therefore, RNA viral attack upon dsRNA sensing occurring at mitochondria could trigger mitochondrial dysfunction that causes mtDNA stress and subsequent mtDNA release in response.

### Release During Bacterial Infection

In addition to viruses, mtDNA release occurs during bacterial infection (Table 1), strengthening the role for mtDNA-mediated innate immune signaling in general pathogen defenses. Infection by *Pseudomonas aeruginosa* induces mitochondrial damage and mtROS alongside the release of ox-mtDNA in a BAX-dependent manner that activates the cGAS, TLR9, and NLRP3 and NLRC4 inflammasomes (167, 168). Activation of cGAS by released mtDNA upregulates autophagy, facilitating the removal of damaged mitochondria and suppressing inflammasome signaling (167–169). Deletion of cGAS, therefore, leads to higher cytokine production, increased bacterial loads, and lung injury in mice (168). Chemical inhibition of OGG1, which increases the levels of ox-mtDNA, enhances mtDNA release, cGAS–STING signaling, and apoptosis, improving cellular responses to *P. aeruginosa* infection both in cultured cells and in vivo (169). In a similar vein, *Escherichia coli* O157:H7 infection causes mtDNA release and NLRP3 inflammasome activation, and upregulation of autophagy restrains inflammasome activation by restoring mitochondrial function (170). Release of mtDNA also occurs during *Salmonella Typhimurium* infection, and mtDNA–cGAS–STING signaling inhibits bacterial replication (171). Certain strains of *Mycobacterium tuberculosis* also trigger mtDNA–cGAS–STING signaling (172). Mitochondrial dysfunction exacerbates inflammatory responses to *M. tuberculosis*, as macrophages with the PD disease mutation *Lrrk2*^G2019S^ exhibit mitochondrial dysfunction, enhanced mtDNA release, and AIM2 inflammasome activation upon infection. Increased mtROS production in *Lrrk2*^G2019S^ macrophages drives the localization of gasdermin D to mitochondria, enhancing mitochondrial DAMP release and ultimately causing necroptosis (173). *Mycobacterium abscessus* infection also causes mitochondrial dysfunction and increased mtROS, which aids in pathogen escape from the phagosome but also causes ox-mtDNA release leading to cGAS and NLRP3 inflammasome activation (174). These studies highlight that, while mtDNA–innate immune signaling is important for combating bacterial pathogens, overactivation of mtDNA-driven signaling can contribute to harmful inflammation and tissue injury during the course of infection.

### Release in Aging and Senescence

Two broadly accepted hallmarks of aging are mitochondrial damage and dysfunction (175) and chronic, low-grade inflammation (176). The now well-established role of mitochondria in innate immune signaling and inflammation begs the question: To what degree are these two aging hallmarks connected? The answer is likely not simple, since mitochondria have both negative (e.g., loss of energetic and metabolic capacity, promotion of ROS-mediated oxidative stress) and positive (e.g., adaptive/mitohormetic signaling) roles in aging and longevity (175, 177). Here, we focus on a potentially negative role of mtDNA release as a driver of age-related inflammation, the evidence for which (pro or con) is only beginning to amass, especially in humans. The hypothesis that CCF-mtDNA induces age-associated inflammation with systemic effects on many tissues is supported by the observation that it increases with age in a Caucasian cohort study, with a significantly increased rate after age ~50 (178). Furthermore, circulating inflammatory cytokines were highest in those individuals with the highest CCF-mtDNA. CCF-mtDNA also correlated with serum GDF15, a biomarker of mitochondrial stress that may also play an anti-inflammatory role (179). Interestingly, the amount of CCF-mtDNA correlates with age-related neutrophil phenotypes (180), suggesting it may be involved in age-related dysfunction of the immune system. The relevant form of CCF-mtDNA in circulation (membrane-free versus membrane-encapsulated) is also important to consider since it has been reported that the level of mtDNA in EVs actually decreases with age (181).

There is some evidence that intracellular mtDNA release also promotes age-related phenotypes (182). Oxidative mitochondrial and mtDNA damage have long been implicated in age-related macular degeneration (183). Recently, reduced dicer expression and increased Alu–RNA were shown to cause mtDNA release, amplified cGAS–STING signaling, and noncanonical inflammasome activation that may contribute to this disease (106, 184). Similarly, melatonin decreases with age and its deficiency causes defects in mitochondrial homeostasis, increased mtDNA release, and a complex inflammatory phenotype in mouse neurons (185). This study also reported increased mtDNA-release-mediated inflammation in Huntington’s disease model mice that is rescued by exogenous melatonin. In aged mouse macrophages, mtDNA-release-mediated cGAS–STING signaling occurs downstream of defects in mitophagy due to a combination of deficient PINK1–PARKIN activity and impaired lysosome biogenesis, which may contribute to liver inflammation (186). Age-dependent increases in mtDNA-release-mediated cGAS–STING–IRF3 signaling may also contribute to age-related hearing loss (187). In addition, the progeroid, proofreading-deficient Polγ (mtDNA mutator) mouse has increased mtDNA release and ISG expression in macrophages that are STING dependent, as well as elevated CCF-mtDNA in plasma that drives type 1 IFN signaling and systemic inflammation (188).

Cellular senescence is another hallmark of aging and is linked to chronic inflammation through the senescence-associated secretory phenotype (SASP) (189). It is already appreciated that cytosolic chromatin fragments can drive cellular senescence and the SASP via cGAS–STING signaling (66, 67). Mitochondrial dysfunction, ROS, and innate immune signaling (e.g., DNA sensing through cGAS–STING) are also known to contribute to senescence and the SASP, and these are even linked through a retrograde JNK signaling pathway (190–193). While the involvement of mtDNA-release-mediated inflammation in senescence and the SASP is not yet clear, there is some evidence that it might play a role. In late-generation *Terc*^−/−^ mice, which lack telomerase activity, macrophages with short telomeres exhibited a senescence phenotype and had increased mtDNA-release-mediated cGAS–STING signaling upon influenza infection that might contribute to enhanced viral pneumonia susceptibility with age (194). In aged mice, senescent pancreatic β cells accumulate and exhibit mtDNA-release-mediated cGAS–STING signaling that is exacerbated by a high-fat diet and associated with a SASP phenotype (92). Release of mtDNA also appears to promote senescence in lung fibroblasts and alveolar endothelial cells from idiopathic pulmonary fibrosis patients, as well as in control cells, when senescence was induced, all of which is cGAS dependent (195, 196). These results suggest that mtDNA-release-mediated inflammation is involved in this age-related disease. Passos and colleagues (197) recently found that minority-MOMP induces mtDNA release through BAK/BAX pores and mediates cGAS–STING signaling during cellular senescence, and inhibiting this in vivo decreases inflammatory markers and improves health span measures in mice. Thus, it is becoming clear that mtDNA-mediated inflammation is involved in aging and age-related pathology. The well-documented damage and decline in function of mitochondria with age (175), which is accompanied by alterations in mitochondrial dynamics, mtDNA damage, ROS generation, and decreased autophagy and mitophagy due to lysosomal dysfunction (and other factors) (198), could be a perfect storm for enhanced mtDNA release driving age-related chronic inflammation. More studies testing the validity of this intriguing mitoflammation (42) hypothesis in aging are certainly warranted, which likely will involve other mitochondrial DAMPs in addition to mtDNA (e.g., TFAM, N-formyl peptides, cardiolipin, and mtRNA) (8, 58).

### Release in Disease Pathology

Twenty years ago, it was reported that injecting mtDNA into mouse joints caused inflammation and arthritis, while injecting nuclear DNA did not. This was due to ox-mtDNA, and the authors also found increased CCF-mtDNA in the synovial fluid of patients with rheumatoid arthritis (33, 199). Zhang et al. (200) subsequently showed that intraperitoneal-injected mtDNA in rats can quickly enter the circulation, where it could have systemic effects. These early observations were the tip of the iceberg, as it is now known that mtDNA is involved in inflammatory pathology due to the activation of multiple innate immune pathways downstream of its intracellular and extracellular release. Extracellular CCF-mtDNA enters plasma, serum, synovial fluid, and cerebrospinal fluid by mechanisms that remain unclear but are important in terms of systemic inflammation (46), the paracrine effects of which may involve complexes with IL-26 (201). In Table 1, we summarize the literature reporting the role of released mtDNA in inflammatory disease pathology and infection. This highlights the need for standardized methods to detect cytoplasmic mtDNA and CCF-mtDNA for research and clinical applications (202, 203).

## BREAKING THE CODE: WHAT ARE THE REQUIREMENTS FOR MITOCHONDRIAL DNA RELEASE AND INNATE IMMUNE RECOGNITION?

As a relatively nascent field, there are many remaining questions regarding the mechanisms and biochemistry of mtDNA release. First, concerning intracellular release, the BAK/BAX and VDAC pore-mediated mechanisms were first on the scene. At first glance, it seems easy to conclude that whole nucleoids, which are not oxidized, are released by BAK/BAX pores and that this occurs mostly under apoptotic or sub-apoptotic (i.e., minority-MOMP) conditions. And, furthermore, that this is mainly a mechanism for cGAS–STING activation. Likewise, the mPTP–VDAC pore mechanism might easily be slotted into the fragmented, ox-mtDNA release category that is more or less specific for NLRP3 inflammasome activation. The dogma is that TLR9 is primarily a DNA sensor that responds to mtDNA because of its paucity of CpG methylation. While perhaps a good starting point, this is not a sufficient framework to explain even the current literature. For example, as we discuss in the section titled Mechanisms of Mitochondrial DNA Release, (*a*) there are clearly other release mechanisms that are independent of BAK/BAX and mPTP/VDAC, for which the requirements and exact form of mtDNA released are not yet documented, (*b*) multiple innate immune sensors can be activated simultaneously, suggesting that multiple forms of mtDNA, alone or in combination with other DAMPs, are at play, and (*c*) the same perturbation can lead to different mtDNA-release-mediated immune responses (Figure 1) and pathology in different cell and tissue types (153). So, there is still much to be learned about which stresses cause the release of which mtDNA species and why they activate one innate immune sensor versus another versus multiple sensors at the same time.

The physical state of released mtDNA is certainly a major determinant of innate immune recognition. While its oxidation state and intactness (fragmented or whole nucleoids) are key factors, whether it is bound by proteins or encapsulated in membranes is equally important. For example, because cGAS multimerizes when it recognizes DNA, longer DNA substrates can facilitate cGAS activation (69). For the same reason, proteins that induce bends in DNA (e.g., HMG-box proteins like TFAM) aid the presentation of DNA to cGAS for optimal activation, although high TFAM:DNA ratios prevent cGAS activation, presumably due to over-compaction of DNA (69). Therefore, TFAM-bound but not fully compacted mtDNA nucleoids might represent ideal substrates for cGAS. In terms of oxidation, the field has converged on 8-oxoguanine as a key aspect of ox-mtDNA for innate immune recognition, mostly due to observed effects of manipulating the BER enzyme OGG1 that removes this common oxidative mtDNA damage lesion. However, there are additional BER glycosylases in mitochondria, and many other types of oxidative damage can occur in DNA to the purines, pyrimidines, and deoxyribose backbone. Thus, the oxidation code for innate immune recognition may be much more complex. Lastly, the primary sequence requirements for mtDNA innate immune recognition are largely unexplored. Many studies have probed the D-loop region of mtDNA and concluded that it is preferentially released. The exact role of this stable three-stranded region of mtDNA (9) remains a mystery in the field. It is intriguing to consider that it may be part of a specialized cellular stress and immune-sensing mechanism. However, it instead may be the case that the increased steady-state abundance of the D-loop strand itself (as part of the stable three-stranded DNA structure) relative to other mtDNA sequences leads to its preferential detection by PCR or other methods used to assay mtDNA release.

We speculate that each state of mtDNA (described in the paragraph above), when released, is indicative of a certain type of mitochondrial, mtDNA, or cellular stress (Figure 2) that is being detected by the various innate immune receptors to initiate a cognate response (Figure 1). For example, this could be mtDNA stress due to oxidative damage, defective mtDNA replication or repair, or impaired mtDNA segregation during fission. There are also multiple points allowing for the degradation of mtDNA as mitochondria or mtDNA-containing vesicles progress through the mitophagy and autophagy pathways, ultimately fusing with lysosomes to dispose of cargo. Thus, one function of different innate immune sensors might be to determine which step of mtDNA replication or degradation is going awry. Altered mitochondrial and nucleoid morphology are also commonly observed under circumstances when mtDNA is released. For example, elongated mitochondria and enlarged or clustered nucleoids can be indicative of a defect in mtDNA replication or segregation that causes a delay in fission (42, 80, 204). However, many factors can cause changes in mitochondrial and nucleoid morphology (18), and hence these types of morphological indices are not necessarily predictive of the activation of any particular mtDNA release mechanism or innate immune signaling pathway. In addition, there are also mechanisms to prevent or reduce the immunogenicity of mtDNA once released, including degradation by nucleases and deamination by the APOBEC3 enzyme (EC 3.5.4.1) (205). However, one of the main nucleases likely responsible for this, TREX1, cannot efficiently degrade ox-DNA, providing one potential explanation for how released ox-mtDNA can accumulate and signal (206). Clearly, we are far from fully understanding why, when, and how mtDNA is released (inside the cell, outside the cell, and into circulation) and what determines which innate immune sensor is activated under different physiological and pathological circumstances. Breaking this code will indeed be a very exciting area of biochemistry at the interface of cell biology and immunology.

## Figures and Tables

**Table 1:** Inflammatory pathology associated with mtDNA release

Category	Disease	mtDNA-driven inflammatory signaling
Age-related diseases	Age-related hearing loss	■ Increased cytosolic mtDNA, cGAS–STING signaling, and cytokines in the cochlea, inferior colliculus, and auditory cortex of aged C57BL/6J male mice (187).
	Age-related macular degeneration	■ Release of mtDNA leading to activation of cGAS, mitochondrial damage, and mPTP activation, promoting the activity of NLRP3 inflammasomes (106).
Autoimmunity	Periodic fever syndromes	■ Defective autophagy prevents clearance of damaged mitochondria, causing mtROS and mtDNA–NLRP3 inflammasome signaling and hypersecretion of IL-1β and IL-18 in monocytes (207).
	Rheumatoid and inflammatory arthritis	■ Ox-mtDNA in the synovial fluid of patients (199). ■ Injection of mtDNA into joints induces arthritis via NF-κB signaling and recruitment of monocytes and macrophages (33).
	Systemic lupus erythematosus	■ Ox-mtDNA is a component of NETs and triggers type I IFN when released (50–52). ■ IFNα impairs autophagy and leads to an accumulation of dysfunctional mitochondria and cytosolic mtDNA, which drives cGAS–STING signaling (208). ■ Impaired mitophagy in red blood cells prevents the clearance of mitochondria. When these red blood cells are internalized by macrophages, mtDNA is released and drives cGAS–STING signaling (154). ■ Inhibiting VDAC oligomerization ameliorates mtDNA release and lupus-like symptoms in an SLE mouse model (31).
	Systemic sclerosis	■ Patients with ATAD3A mutations display increased type I IFN signaling associated with systemic sclerosis and other symptoms, including neurological. Patient cells display increased ISG expression dependent upon cGAS, STING, and mtDNA, which can be partially reversed by VDAC inhibition (DIDS) or rapamycin treatment (76).
Bacterial infection	*Escherichia coli* O157:H7	■ Mitochondrial damage leading to mtDNA release and NLRP3 inflammasome activation (170).
	*Mycobacterium tuberculosis*	■ Infection by some strains induces mitochondrial stress and ROS, mtDNA release, and cGAS–STING signaling (172).
	*Mycobacterium abscessus*	■ Infection induces mtROS, release of ox-mtDNA, cGAS–STING signaling and NLRP3 inflammasome signaling. mtROS damages phagosomes, enabling bacterial escape and replication (174).
	*Pseudomonas aeruginosa*	■ Infection causes oxidative damage and release of mtDNA fragments from lung cells (209). ■ mtDNA–cGAS signaling activates autophagy, promoting clearance of bacteria as well as damaged mitochondria. Enhanced mtDNA release improves cellular response to infection, and cGAS deficiency impairs bacterial clearance and leads to heightened mtDNA-driven TLR9 and NLRP3/NLRC4 inflammasome responses (167–169).
	*Salmonella Typhimurium*	■ Release of mtDNA and mtDNA–cGAS–STING signaling inhibits bacterial replication (171).
	*Streptococcus pneumoniae*	■ Sepsis induces mtROS, mtDNA damage and release, and activation of TLR9 in heart tissue (210).
Blood disorders	Sickle cell anemia	■ Sickle red blood cells retain mitochondria, leading to an increase in circulating mtDNA, which then triggers increased NET formation dependent upon TBK1 (211).
Cancer	Chemotherapeutic resistance	■ Chronic mtDNA stress causes upregulation of a specific subset of ISGs (IRDs) via the U-ISGF3 complex, promoting enhanced nuclear DNA repair. The chemotherapeutic drug doxorubicin causes mtDNA damage and release, and mtDNA release and signaling in melanoma cells promotes resistance to doxorubicin in vivo (212).
Cardiovascular disease	Atherosclerosis	■ Release of ox-mtDNA and activation of the NLRP3 inflammasome, leading to increased atherosclerotic plaques (110). ■ mtDNA that escapes autophagy activates TLR9 and drives aortic inflammation and atherosclerosis in LDL receptor knockout mice (213). ■ LL-37-bound mtDNA escapes autophagy, accumulates in atherosclerotic plaques, and activates TLR9, causing immune cell activation, recruitment, and autoimmune responses (145). ■ Loss of DNMT3A or TET2 activity in human monocyte-derived macrophages and atherosclerotic macrophages causes downregulation of TFAM, triggering mtDNA stress and release and cGAS–STING signaling (214).
	Cardiomyopathy	■ mtDNA that is not degraded by autophagy, due to depletion of DNase II, drives TLR9 signaling, leading to heart failure (43). ■ Increased cGAS–STING signaling in diabetic cardiomyopathy, partial reversal of pathology with STING inhibition. Palmitic acid treatment of H9C2 cells increases ROS and mtDNA release, suggesting that lipotoxicity drives mtDNA–cGAS–STING signaling (93, 94). ■ Several lines of evidence implicate increased circulating mtDNA and TLR9 signaling in acute myocardial infarction (215).
	Friedreich ataxia	■ Acute knockdown of frataxin in iPSC-derived cardiomyocytes induces mitochondrial dysfunction, mtDNA release, cGAS activation, and a type I IFN response, implicating mtDNA-driven inflammation in cardiomyopathy associated with this disease (216).
	Hypertension	■ Increased circulating mtDNA in hypertensive rats, associated with diminished DNase activity and TLR9 signaling. TLR9 inhibition reduced blood pressure in hypertensive rats, whereas administering a TLR9 agonist to healthy rats increased systolic blood pressure (217).
Chronic inflammatory diseases	Kawasaki disease	■ Increased circulating mtDNA and expression of CD36 and AIM2 (218).
	Idiopathic pulmonary fibrosis	■ Increased mtDNA–cGAS–STING signaling driving senescence in alveolar epithelial cells (195, 196).
	Inflammatory bowel disease	■ Increased circulating mtDNA and TLR9 signaling associated with mitochondrial damage (219).
Dental disease	Pulpitis	■ BAX-mediated mtDNA release occurs during gasdermin-D-driven pyroptosis, activating STING-driven inflammation in odontoblasts (28).
Liver diseases	Acute liver injury	■ Increased levels of circulating mtDNA in response to liver injury with inflammation dependent upon TLR9 (220).
	Hepatocellular carcinoma	■ Hypoxia increases mtROS and mtDNA release; released mtDNA binds HMGB1 and activates TLR9 signaling, which promotes tumor growth (144).
	NASH, NAFLD, and liver fibrosis	■ In the context of HFD-induced NASH, ox-mtDNA is released into plasma and present within microparticles, which activate TLR9. Blocking TLR9 signaling pharmacologically prevents the development of NASH (221). ■ Increased circulating mtDNA associated with fibrosis, NAFLD, and NASH (222).
Metabolic disease	Obesity	■ mtDNA release and cGAS–STING signaling in the adipose tissue of HFD-fed mice. Loss of DsbA-L, a chaperone in the matrix, causes mtDNA release and cGAS–STING signaling, leading to decreased thermogenesis and energy expenditure, thereby promoting obesity. Overexpression of DsbA-L protects against HFD-induced obesity (95, 96). ■ Release of mtDNA into the cytoplasm of pancreatic beta cells accelerates senescence, causing glucose intolerance and insulin resistance in aged, HFD-fed mice, and these effects are improved by STING inhibition (92).
	Type II diabetes	■ Increased circulating mtDNA, leading to AIM2 inflammasome activity and secretion of IL-1β and IL-18 (223).
Neurodegenerative disease	Amyloid lateral sclerosis	■ Disease mutations in TDP-43 cause its localization to mitochondria, where it triggers mtDNA release and cGAS–STING signaling dependent upon VDAC and mPTP but not BAK/BAX. Deletion of STING in a TDP-43 overexpression mouse model of ALS slows neurodegeneration and extends life span (224).
	Huntington’s disease	■ Increased mtDNA release that correlates with disease progression and is associated with ox-mtDNA in synaptic mitochondria. Increased cGAS–STING signaling associated with cytokine production and synaptic degeneration. ■ Melatonin administration reduces mtDNA release and cGAS–STING signaling in mouse models of HD. ■ Melatonin deficiency is associated with increased mtROS, mtDNA damage, and permeability transition, causing mtDNA–cGAS–STING signaling, and is associated with accelerated aging (185).
	Parkinson’s disease	■ mtDNA mutational stress increases circulating mtDNA and cytokines and causes dopaminergic neurodegeneration in a STING-dependent fashion in *Parkin^−/−^* and *mutator* mice (5). ■ Escape of mtDNA from lysosomes, causing cell death in cultured cells and zebrafish models of PD (6). ■ Loss of VPS13C/PARK23, which is mutated in PD, causes lysosomal dysfunction leading to mtDNA leakage into the cytosol, cGAS–STING signaling, and impaired STING degradation, which perpetuates signaling (4). ■ Deficiency of LRRK2, also mutated in PD, leads to increased mitochondrial stress and mtDNA–cGAS–STING signaling. *Lrrk^−/−^* mice display higher inflammatory responses when infected with *M. tuberculosis* (7). The disease mutation *Lrrk2*^G2019S^ causes mitochondrial dysfunction, causing enhanced AIM2 inflammasome activation in macrophages infected by *M. tuberculosis*. Enhanced mtROS production drives the association of gasdermin D with mitochondria, exacerbating mitochondrial DAMP release and promoting cell death via necroptosis as opposed to pyroptosis (173).
Trauma and injury	Acute lung injury and acute respiratory distress syndrome	■ Increased levels of circulating mtDNA that correlate with disease severity, along with increased levels of mtDNA present in the bronchoalveolar lavage fluid and heightened TLR9, cGAS–STING, and NLRP3 inflammasome signaling (225).
	Acute kidney injury	■ Cisplatin induces cGAS–STING signaling during acute kidney injury; cultured cells treated with cisplatin release mtDNA in a BAX-dependent manner (27). ■ mtDNA is released into circulation during septic acute kidney injury and activates TLR9 signaling, causing renal dysfunction (226). ■ Increased IL-18, suggesting that circulating mtDNA may also drive inflammasome activation (227). ■ Increased circulating mtDNA and TFAM arising from acute kidney injury drive inflammation and mitochondrial dysfunction in secondary lung injury (228).
	Burn trauma	■ Release of mtDNA into circulation and cGAS–STING signaling in unaffected tissue, secondary acute lung injury associated with activated neutrophils (229).
	Sepsis	■ Increased circulating mtDNA, and higher levels correlate with increased mortality (230).
	SIRS	■ mtDNA is released into circulation and activates TLR9, leading to cytokine production and neutrophil activation (48, 49). ■ Increased circulating mtDNA after severe injury, and higher levels correlate with SIRS, MODS, and mortality (230, 231).
Viral infection	Many viruses	■ The following cause mtDNA release during infection: HSV-1 and HSV-2 (65), IAV (157, 119), ECMV (157), DENV (158, 159), ZIKV (161), MeV (162), SARS-CoV-2 (164), KSHV (232), SFTSV (120), PRRSV (160).

Abbreviations: ALS, amyloid lateral sclerosis; cGAS, cGMP–AMP synthase; DAMP, damage-associated molecular pattern; DENV, dengue virus; DIDS, 4,4’-Diisothiocyanatostilbene-2,2’-disulfonate; ECMV, encephalomyocarditis virus; HD, Huntington’s disease; HFD, high-fat diet; HSV, herpes simplex virus; IAV, influenza A virus; IFN, interferon; IL, interleukin; ISG, interferon-stimulated gene; iPSC, induced pluripotent stem cells; KSHV, Kaposi’s sarcoma- associated herpesvirus; LDL, low-density lipoprotein; MeV, measles virus; mPTP, mitochondrial permeability transition pore; mtDNA, mitochondrial DNA; MODS, multiple organ dysfunction syndrome; NASH, nonalcoholic steatohepatitis; NAFLD, nonalcoholic fatty liver disease; NET, neutrophil extracellular traps; NF-κB; nuclear factor κB; NLRP3, NOD-, LRR-, and pyrin domain-containing protein 3; ox-mtDNA, oxidized mtDNA; PD, Parkinson’s disease; PRRSV, porcine reproductive and respiratory syndrome virus; ROS, reactive oxygen species; SARS-CoV-2, severe acute respiratory syndrome coronavirus 2; SFTSV, severe fever with thrombocytopenia syndrome virus; SIRS, systemic inflammatory response syndrome; SLE, systemic lupus erythematosus; TBK1, TANK-binding kinase 1; TFAM, transcription factor A mitochondrial; TLR, Toll-like receptor; VDAC, voltage-dependent anion channel; ZIKV, Zika virus.
